# Tissue Oxygenation in Response to Different Relative Levels of Blood-Flow Restricted Exercise

**DOI:** 10.3389/fphys.2019.00407

**Published:** 2019-04-11

**Authors:** Joana F. Reis, Pedro Fatela, Goncalo V. Mendonca, Joao R. Vaz, Maria J. Valamatos, Jorge Infante, Pedro Mil-Homens, Francisco B. Alves

**Affiliations:** ^1^Laboratory of Physiology and Biochemistry of Exercise, Faculdade de Motricidade Humana, Universidade de Lisboa, Lisbon, Portugal; ^2^Ciper, Faculdade de Motricidade Humana, Universidade de Lisboa, Lisbon, Portugal; ^3^Universidade Europeia, Lisbon, Portugal; ^4^Neuromuscular Research Lab, Faculdade de Motricidade Humana, Universidade de Lisboa, Lisbon, Portugal; ^5^Biomechanics and Functional Morphology Laboratory, Faculdade de Motrocidade Humana, Universidade de Lisboa, Lisbon, Portugal; ^6^Department of Biomechanics, University of Nebraska at Omaha, Omaha, NE, United States; ^7^Spertlab, Faculdade de Motricidade Humana, Universidade de Lisboa, Lisbon, Portugal

**Keywords:** muscle oxygenation, KAATSU, resistance exercise, near-infrared spectroscopy, oxygen extraction

## Abstract

Blood flow restrictive (BFR) exercise elicits a localized hypoxic environment compatible with greater metabolic stress. We intended to compare the acute changes in muscle microvascular oxygenation following low-intensity knee extension exercise, combined with different levels of BFR. Thirteen active young men (age: 23.8 ± 5.4 years) were tested for unilateral knee extension exercise (30 + 15 + 15 + 15 reps at 20% one repetition maximum) on four different conditions: no-BFR (NOBFR), 40, 60, and 80% of arterial occlusion pressure (AOP). Deoxyhemoglobin+myoglobin concentration Deoxy[Hb+Mb], total hemoglobin [T(H+Mb)] and tissue oxygen saturation [TOI] were measured on the vastus lateralis muscle using near-infrared spectroscopy (NIMO, Nirox srl, Brescia, Italy). The magnitude of change in Deoxy[Hb+Mb]during exercise was similar between 60 and 80% AOP. Overall, compared to that seen during 60 and 80% AOP, NOBFR as well as 40% AOP resulted in a lower magnitude of change in Deoxy[Hb+Mb] (*p* < 0.05). While the oxygen extraction decreased during each inter-set resting interval in NOBFR and 40% AOP, this was not the case for 60 or 80% AOP. Additionally, TOI values obtained during recovery from each set of exercise were similarly affected by all conditions. Finally, our data also show that, when performed at higher restrictive values (60 and 80%), BFR exercise increases total Deoxy[Hb+Mb] extraction (*p* < 0.05). Taken together, we provide evidence that BFR is effective for increasing deoxygenation and reducing tissue oxygenation during low-intensity exercise. We also showed that when using low loads, a relative pressure above 40% of the AOP at rest is required to elicit changes in microvascular oxygenation compared with the same exercise with unrestricted conditions.

## Introduction

The chronic effect of using tourniquet cuffs to restrict muscle blood flow during resistance exercise on muscle size and strength are well established in the available literature (Loenneke et al., 2012; Slysz et al., 2016). Despite significant efforts to fully comprehend the physiological basis of muscle growth resulting from blood flow restricted (BFR) exercise, the specific mechanisms underlying such response remain largely unknown (Manini and Clark, 2009). There is general agreement that muscle hypertrophy is triggered by the combined effect of mechanical tension, muscle damage and metabolic stress (Schoenfeld, 2010). Several studies have shown that performing BFR exercise with 20% of 1RM induces the same or even greater fatiguing stimulus when compared with high intensity exercise training (Takarada et al., 2000; Cook et al., 2007). Thus, it can be used in multiple exercise settings, particularly when high intensity resistance exercise is not recommended (Cook et al., 2007). Since BFR exercise is typically performed using light loads, metabolic stress is believed to be largely responsible for enhancements in both muscle size and strength after training (Takarada et al., 2000, 2002).

Past research has shown that microvascular oxygenation varies as a function of BFR absolute pressure and duration (Karabulut et al., 2014; Neto et al., 2014). Nevertheless, it is important to note that BFR exercise is typically structured using a predetermined number of sets and repetitions and that, within this context, the magnitude of neuromuscular activation and fatigue varies as a function of BFR relative pressure (Fatela et al., 2016). For this reason, several reports focused on exploring the overall impact (i.e., during and between sets) of BFR on microvascular oxygenation during low-intensity resistance exercise (Ganesan et al., 2015; Lauver et al., 2017; Yanagisawa and Sanomura, 2017). Their findings are somewhat conflicting, and this is probably a consequence of differences between methodological designs (e.g., exercise selection, BFR level as well as duration, contraction mode and site for monitoring tissue oxygenation). To our knowledge, only one previous investigation examined the role of relative BFR pressure on microvascular oxygenation (Kilgas et al., 2018). Using handgrip exercise, it was shown that setting BFR to 60 and 80% of the pressure required to block arterial blood flow, or arterial occlusion pressure (AOP) elicited a reduction in tissue saturation index and an increase in deoxyhemoglobin+myoglobin concentration (Deoxy[Hb+Mb]). However, due to the characteristics of this specific exercise paradigm, these findings may not be extensive to dynamic exercise involving larger muscle mass. Determining the optimal level of BFR pressure, relative to AOP, is fundamental for maximizing the effectiveness of exercise prescription because it might influence the possible mechanism for the adaptations following BFR training (Cayot et al., 2014; Scott et al., 2015).

Therefore, we aimed at comparing the acute response of Deoxy[Hb+Mb], total hemoglobin+myoglobin [T(H+Mb)] and tissue oxygen saturation (TOI) in the vastus lateralis muscle during low-intensity exercise performed at different levels of relative BFR. We hypothesized that, during dynamic knee extension exercise, the peripheral deoxygenation would increase, and total hemoglobin+myoglobin would increase as a function of the percent arterial occlusion pressure.

## Materials and Methods

### Participants

Thirteen active young men (age: 23.8 ± 5.4 years; height: 174.8 ± 4.2 cm; body mass: 69.8 ± 7.0 kg; Systolic blood pressure: 122.6 ± 7.0 mmHg; diastolic blood pressure: 76.7 ± 8.5 mmHg; AOP: 136.6 ± 9.3 mmHg) volunteered to participate in this study. The participants were fully informed of any risk and discomfort associated with the experiments before providing written consent. Participants were not enrolled in any kind of resistance or endurance training in the 6 months prior to the participation in the study. They were all non-smokers and free from any known cardiovascular and metabolic diseases, as assessed by medical history. The participants were instructed to maintain the same level of physical activity throughout the course of the study. They were asked to avoid exercise as well as the intake of caffeine and alcohol for at least 24 h before testing. Testing was performed at the same time of day for standardization purposes with at least 48 h between testing sessions. This study was approved by the Faculty’s Ethics Committee (CEFMH 17/2014) and in accordance with the Declaration of Helsinki.

### Experimental Design

All participants were familiarized with the testing procedures in two separate sessions. Metronome pacing and AOP measurements were performed during the first session. Then, on a different day, all participants performed a baseline session, where (a) AOP for the right lower limb was reassessed, (b) one repetition maximum (1RM) was determined for the right knee extensors and (c) testing (including metronomic pacing) was again reproduced. Using a crossover design, participants then visited the laboratory on four additional days to complete the following resistance exercise trials in a randomized order: without BFR (NOBFR), 40, 60, and 80% AOP. Participants were tested in the seated position and were fixed with chest and abdominal straps. Vascular restriction was elicited using a 13 × 124 cm pneumatic cuff (SC12L Tourniquet Cuffs, D. E. Hokanson, Inc., Bellevue, WA), applied to the most proximal portion of the right thigh and the pressure was maintained throughout the test.

The exercise protocol was performed using an isokinetic dynamometer (Biodex System 3, Biodex Medical Systems, Shirley, NY), and consisted of a series of unilateral knee extensions at 20% of 1RM throughout all testing sessions. This training intensity was selected because of its effectiveness in inducing fatigue when combined with BFR (Takarada et al., 2000; Cook et al., 2007). During exercise, participants were monitored for microvascular oxygenation using near-infrared spectroscopy (NIRS) (NIMO, Nirox srl, Brescia, Italy). Knee-extension peak torque was also determined during maximal voluntary isometric contraction (MVIC) before and after acute exercise.

### Determination of AOP

Arterial occlusion pressure was determined using a vascular Doppler probe (PD1+ Combi, Ultrasound Technologies Ltd., Caldicot, United Kingdom) placed over the right posterior tibial artery, halfway between the posterior border of the medial malleolus and the Achilles tendon. A pneumatic cuff was inflated (E20/AG101 Rapid Cuff Inflator) gradually up to the point when the auscultatory pulse of the posterior tibial artery was interrupted (Fatela et al., 2018). To guarantee similar cuff placement between familiarization and testing sessions, a photographic record was made for each participant.

### 1RM Testing

The maximal torque produced in a single repetition was determined for the right knee extension using the isotonic mode of the isokinetic dynamometer. The participants were asked to complete one repetition through a full range of motion (90°). Strong verbal encouragement was given in each trial, and 2 min of recovery were allowed between attempts. 1RM was always determined within five trials (mean value: 219.2 ± 37.8 Nm).

### Maximal Voluntary Isometric Contraction

Maximal voluntary isometric contraction was determined at the optimal joint angle for right knee extension in all testing sessions. Participants performed three isometric knee extension trials (3 s per trial). They were instructed to exert their maximum force as fast and hard as possible. One min of recovery between trials was allowed. Peak torque was set as pre- and post-exercise MVIC (2 min post-cuff deflation).

### Exercise Protocol

After a standardized warm-up (6 min of unloaded cycle-ergometry), participants performed 4 sets of knee extension at 20% 1RM (30 + 15 + 15 + 15 reps, set 1–4, respectively), with 30 s of passive rest between sets (rest 1–3) (Yasuda et al., 2008; Loenneke et al., 2013). A metronome was used to control the concentric–concentric mode, with 1 s for knee extension (20% 1-RM) and another for knee flexion (unloaded). Verbal encouragement was provided to warrant that each participant completed the full exercise protocol. For safety reasons, a pulse oximeter (Onyx^®^ II 9560, Nonin Medical Inc., Plymouth, MN) was placed in the right *hallux*, immediately after each set to ensure that blood flow was not completely halted by tissue edema. In the BFR sessions, the cuff was inflated before exercise.

### NIRS Signal

Near-infrared spectroscopy provides non-invasive information about the changes in oxygenation and hemodynamics in muscle tissue based on the oxygen-dependent characteristics of near-infrared light (Perrey and Ferrari, 2018) and has been validated for multiple forms of resistance exercise (Pereira et al., 2007). NIRS measurements were performed on the vastus lateralis muscle of the right leg throughout the entire duration of the exercise protocol. The skin of each participant’s right leg was initially cleaned and shaved. Then, the probe was placed on the belly of the muscle, midway between the lateral epicondyle and greater trochanter of the femur. The placement of the probe was marked with indelible ink and a photographic recording was taken to ensure similar placement of the probe between sessions. The probe was attached to the skin surface with tape and then covered with an optically dense elastic bandage. This minimized its movement and prevented the intrusion of extraneous light and loss of near NIR-transmitted light from the field of interrogation.

Deoxy[Hb+Mb], oxy hemoglobin+myoglobin [(Hb+Mb)O_2_] and [T(Hb+Mb)] were quantified with a continuous-wave tissue oximeter (NIMO, Nirox srl, Brescia, Italy). TOI was expressed in % and calculated as:

(1) [(Hb+Mb)O2]/[(Hb+Mb)O2]+Deoxy[Hb+Mb] × 100.

Briefly, this system is based on the O_2_ dependency of absorption changes for near infra-red light in hemoglobin and myoglobin and it consists on an emission probe which emits three wave lengths (685, 850, and 980 nm) and a photon detector. The intensity of incident and transmitted light was recorded continuously at 40 Hz and used to estimate Deoxy[Hb+Mb], (1) [(Hb+Mb)O2] and [T(Hb+Mb)](Rovati et al., 2004; Ferrari et al., 2011). As the NIRS signal is unable to distinguish between hemoglobin and myoglobin, results are considered as the combined O_2_ saturation of these metalloproteins. To account for the possible influence of the local fat layer on NIRS, a real-time correction using an algorithm included in the software (Nimo Data Analysis Peak) was used. Deoxy[Hb+Mb]signal is less dependent of changes in blood flow and is considered as an indicator of fractional O_2_ extraction within the microvascular level (Ferrari et al., 1997). [T(Hb+Mb)] reflects the total amount of hemoglobin, and it can be interpreted as changes in blood volume within the tissue vascular beds (Van Beekvelt et al., 2001). TOI reflects the dynamic balance between O_2_ supply and O_2_ uptake and is independent of near-infrared photon path length in muscle tissue (Brocherie et al., 2015).

### Data Handling

Baseline values for each testing session were established as 1-min averages for NIRS derived signal. To minimize the impact of baseline differences between trials, Deoxy[Hb+Mb] and [T(Hb+Mb)] values were computed as changes (Δ) from baseline within each testing session. Deoxy[Hb+Mb] and [T(Hb+Mb)] and TOI were measured throughout the exercise protocol. For these analyses, sets were divided into thirds and mean value ± SD of the last third was used as representative of each correspondent phase. (Set 1, Recovery 1, Set 2, Recovery 2, Set 3, Recovery 3, Set 4). Δ [T(Hb+Mb)] was also analyzed for the recovery periods.

Relative recovery (%Rec) for Deoxy[Hb+Mb] and [T(Hb+Mb)] and TOI was determined as the % difference between the last third of recovery and the last third of the previous set. Total oxygen extraction was computed as the area under the Deoxy[Hb+Mb] curve including all sets and recovery periods (Soares et al., 2017).

### Statistical Analysis

Data are reported as mean ± SD, unless otherwise specified. The Mauchly’s test was used to test the assumption of sphericity. The Greenhouse-Geisser correction was implemented to adjust the degrees of freedom for the averaged tests of significance when the assumption of sphericity was not met. Paired *t-*tests were used for exploring possible differences in peak torque from pre- to post-exercise at each condition (NOBFR, 40, 60, and 80% AOP). A two-way repeated measures ANOVA [four conditions (NOBFR vs. 40 vs. 60 vs. 80% AOP) × 5 times (Pre-exercise vs. set 1 vs. set 2 vs. set 3 vs. set 4)] was used to determine the impact of BFR on each dependent variable (i.e., Deoxy[Hb+Mb] and [T(Hb+Mb)] and TOI). Another two-way repeated measures ANOVA [four conditions (NOBFR vs. 40 vs. 60 vs. 80% AOP) × 3 times (recovery 1 vs. recovery 2 vs. recovery 3)] was additionally used to determine the impact of each condition on the %Rec of Deoxy[Hb+Mb] and [T(Hb+Mb)] and TOI. When a significant effect was detected at a significance level of *p* < 0.05, *t*-tests were used for *post hoc* comparisons. Adjustment for multiple comparisons was made with Bonferroni’s correction. All statistical calculations were computed using the Statistical Package for the Social Sciences (SPSS) version 25.0 (SPSS Inc., Chicago, IL). Significance was set at *p* < 0.05.

## Results

Peak torque was similar between pre- and post-exercise time points in all conditions, except for 80% AOP (reduction of 5.8%, *p* < 0.05). Tables 1–3 show the changes of Deoxy[Hb+Mb] and [T(Hb+Mb)] and TOI in transition from baseline to exercise (sets 1–4). We obtained a condition-by-time interaction for Deoxy[Hb+Mb] and [T(Hb+Mb)] (*F* = 12.4 and 10.3, *p* < 0.05; respectively). *Post hoc* analyses indicate that [HHb] increased from pre-exercise to set 1 in both NOBFR and 40% AOP (*p* < 0.05). No other changes were noted for this parameter during exercise. With the exception of pre-exercise, both these conditions exhibited similar Deoxy[Hb+Mb] values over time. As importantly, Deoxy[Hb+Mb] remained unchanged over time during exercise performed at 60 and 80% AOP. Nevertheless, Deoxy[Hb+Mb] values were consistently lower during NOBFR and 40% AOP when compared to that seen at 60 and 80% AOP throughout all time points (*p* < 0.05). Finally, the acute response of Deoxy[Hb+Mb] to low-intensity exercise was similar between 60 and 80% AOP.

**Table 1:** Delta changes in deoxygenated hemoglobin+myoglobin (AU) (Mean and ± standard deviation) for before exercise (Pre) and Set 1–4 in each blood flow restrictive pressure.

	Pre	Set 1	Set 2	Set 3	Set 4
NOBFR	-2.3 ± 4.9	19.4 ± 8.8^a^	18.9 ± 11.0	19.3 ± 11.3	19.5 ± 11.2
40% AOP	11.0 ± 4.8^∗^	21.2 ± 8.8^a^	20.0 ± 8.3	19.9 ± 8.6	20.2 ± 8.4
60% AOP	18.7 ± 10.9^∗§^	27.3 ± 10.9^∗§^	27.6 ± 11.5^∗§^	27.8 ± 12.0^∗§^	27.3 ± 11.8^∗§^
80% AOP	25.6 ± 10.9^∗§^	27.3 ± 12.1^∗§^	27.7 ± 12.3^∗§^	27.2 ± 12.6	27.5 ± 12.4^∗§^

*^∗^Significantly different from NOBFR; ^§^ Significantly different from 40% AOP; ^*a*^Significantly different from PRE; (*p* < 0.05).*

**Table 2:** Delta changes in total hemoglobin+myoglobin (AU) (Mean and ± standard deviation) for before exercise (Pre) and Set 1–4 in each blood flow restrictive pressure.

	Pre	Set 1	Set 2	Set 3	Set 4
NOBFR	-2.5 ± 13.2	-5.0 ± 14.9	-3.7 ± 15.3	-6.3 ± 16.6	-3.8 ± 18.1
40% AOP	24.1 ± 15.0*	-4.6 ± 16.6a	-4.3 ± 14.2	-4.4 ± 18.6	-0.8 ± 17.8
60% AOP	45.1 ± 24.6*§	6.8 ± 14.1a	12.4 ± 14.0§	13.2 ± 14.6*	13.5 ± 18.0
80% AOP	48.5 ± 23.0*§	-0.8 ± 18.7a	8.5 ± 14.5	6.6 ± 15.8	12.3 ± 13.4

*^∗^Significantly different from NOBFR; ^§^ Significantly different from 40% AOP; ^*a*^significantly different from PRE; (*p* < 0.05).*

**Table 3:** Tissue oxygenation index (AU) (Mean and ± standard deviation) before exercise (Pre) and Set 1–4 in each blood flow restrictive pressure.

	Pre	Set 1	Set 2	Set 3	Set 4
NOBFR	80.6 ± 3.5	65.2 ± 9.5^a^	65.9 ± 11.1	65.0 ± 11.1	65.6 ± 10.2
40% AOP	72.7 ± 7.4	58.1 ± 13.9^a^	59.0 ± 14.4	59.1 ± 14.4	60.4 ± 12.7
60% AOP	73.1 ± 7.5	58.9 ± 14.2^a^	60.5 ± 13.6	61.0 ± 12.6	61.5 ± 12.8
80% AOP	69.7 ± 8.9	55.6 ± 13.8^a^	58.5 ± 13.1	58.3 ± 13.3	59.9 ± 11.9*

*^∗^Significantly different from NOBFR; ^*a*^significantly different from PRE; (*p* < 0.05).*

[T(Hb+Mb)] decreased with BFR exercise (*p* < 0.05) and then remained stable after set 1. Conversely, no changes were seen in [T(Hb+Mb)] in response to NOBFR exercise. There were significant differences in pre-exercise [T(Hb+Mb)] between NOBFR, AOP 40 and 60% (*p* < 0.05). Conversely, this was not the case for comparisons between AOP 60 and 80%. Even though, all conditions attained similar endpoints at the completion of set 1 and 4, [T(Hb+Mb)] was higher in 60 vs. 40% AOP after set 2 and 3 (*p* < 0.05). In Set 3, [T(Hb+Mb)] was also increased following exercise with 60% AOP vs. NOBFR (*p* < 0.05).

There were significant main effects of condition and time for TOI (*F* = 4.3 and 63.0, respectively; *p* < 0.05). Follow up analyses revealed that TOI was lower in 80% AOP than in NOBFR (*p* < 0.05). No other differences were seen between conditions. Additionally, TOI decreased in all conditions from pre-exercise to set 1 (*p* < 0.05) and then remained stable until the end of set 4.

During the inter-set recovery intervals, the relative changes from baseline in [T(Hb+Mb)] presented a significant main effect for condition (*F* = 21.7; *p* < 0.01). Follow up analyses revealed that this variable increased progressively with the restrictive pressures. However, there were no significant differences between 60 and 80% AOP (Table 4).

**Table 4:** Delta changes in total hemoglobin+myoglobin (AU) (Mean and ± standard deviation) for Recovery 1–3 in each blood flow restrictive pressure.

	Recovery 1	Recovery 2	Recovery 3
NOBFR	6.6 ± 10.3	4.3 ± 13.8	4.1 ± 16.2
40% AOP	26.8 ± 13.2	25.0 ± 17.8	25.1 ± 20.5*
60% AOP	43.8 ± 13.0	41.7 ± 12.0	43.4 ± 15.7*§
80% AOP	41.5 ± 17.6	40.9 ± 15.3	45.2 ± 16.9*§

*^∗^Significantly different from NOBFR; ^§^ Significantly different from 40% AOP (*p* < 0.05).*

**FIGURE 1:**
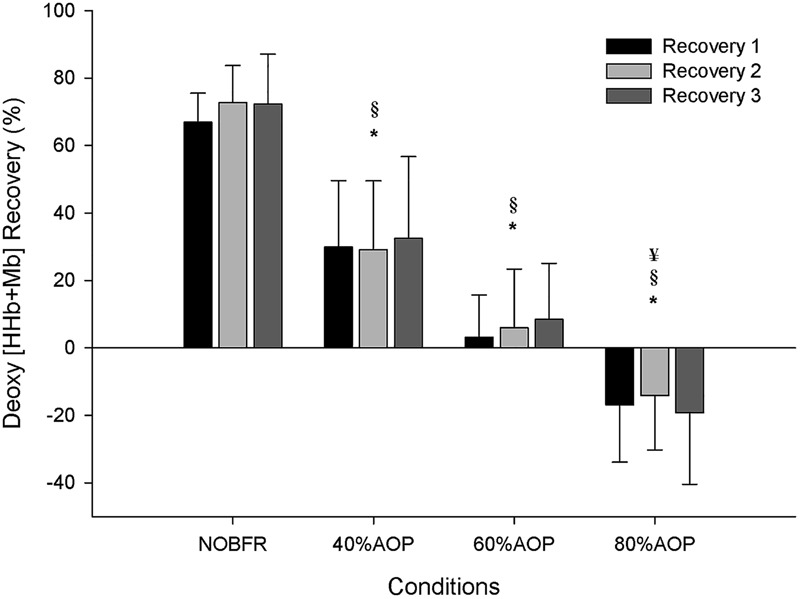
Deoxygenation percentage of recovery during inter-set intervals. ^∗^Significantly different from NOBFR; ^§^ Significantly different from 40% AOP; ^¥^Significantly different from 60% AOP; *p* < 0.05.

**FIGURE 2:**
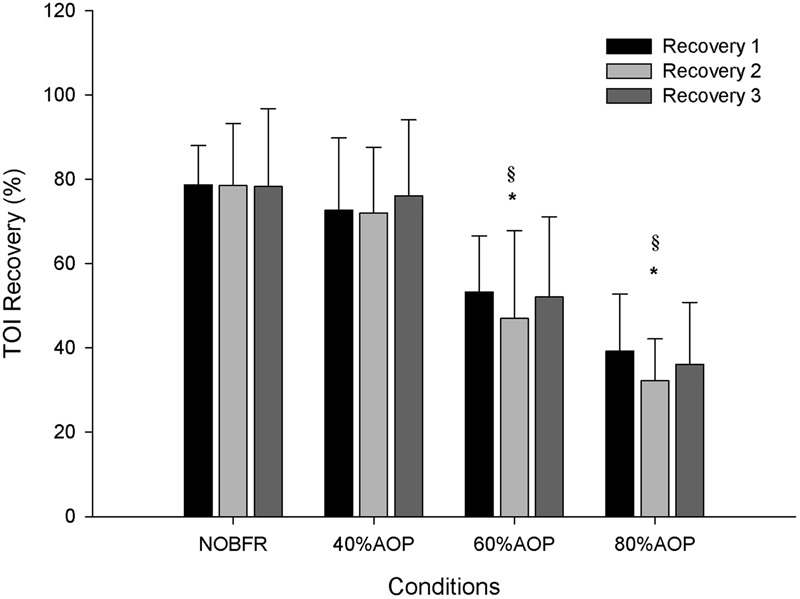
Tissue oxygenation percentage of recovery during inter-set intervals. ^∗^Significantly different from NOBFR; ^§^ Significantly different from 40% AOP; *p* > 0.05.

As depicted in Figure 1, depending on the condition, there were different levels of muscle oxygenation during each inter-set recovery period. Specifically, while Deoxy[Hb+Mb] exhibited a high level of recovery following each NOBFR and 40% AOP exercise set, this was not the case for 60 nor 80% AOP (*p* < 0.05). There was virtually no recovery in Deoxy[Hb+Mb] after 60% AOP and, in 80% AOP, the level of oxygen extraction actually increased. TOI exhibited a significant recovery in all conditions during the inter-set periods (*p* < 0.05). The magnitude of TOI recovery was similar between NOBFR and 40% AOP and between 60 and 80% AOP, respectively (Figure 2). [T(Hb+Mb)] showed no significant differences between conditions for the percentage of recovery from each set.

**FIGURE 3:**
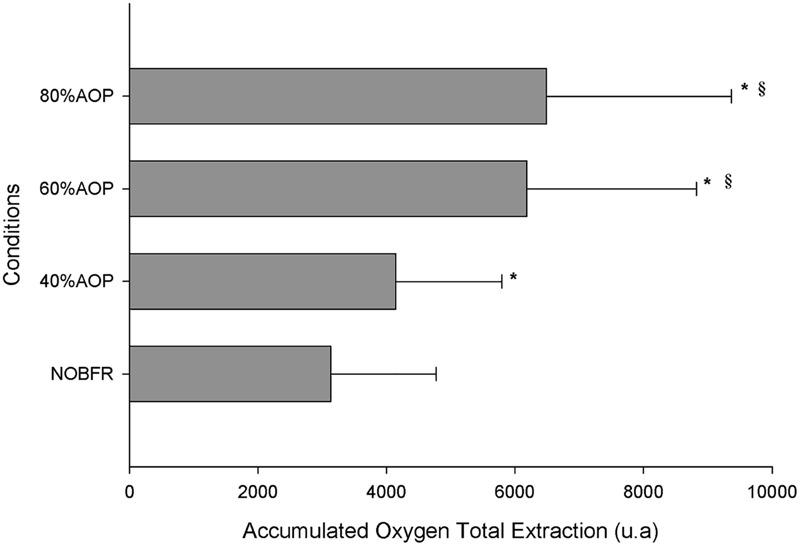
Total oxygen extraction represented as accumulated change from baseline in Deoxy[Hb+Mb] ^∗^Significantly different from NOBFRE; ^§^ Significantly different from 40% AOP; *p* > 0.05.

The area under the Deoxy[Hb+Mb]curve was used to explore the impact of each exercise protocol on total oxygen extraction. As can be seen in Figure 3, the area under the Deoxy[Hb+Mb] curve increased as a function of the percentual AOP pressure used (*p* < 0.05). However, it should be noted that the differences in this parameter did not achieve significance for comparisons between 60 and 80% AOP (*p* > 0.05).

## Discussion

We explored the interaction between different levels of BFR, relative to AOP, and muscle oxygenation in response to an acute multi-set knee-extension exercise protocol. It was demonstrated that BFR increases deoxygenation and hampers tissue oxygenation during low-intensity muscle contractions. Thus, our hypotheses were partially confirmed. However, we also showed that to induce a considerable stress on microvascular oxygenation, represented by increased deoxygenation levels, using low loads (20% RM), BFR should be set above 40% of AOP. Otherwise, the level of muscle oxygenation/deoxygenation is not substantially different from that seen during non-BFR exercise. Since we did not test pressures between 40 and 60% AOP, we can only say that in the three pressures studied, 60% AOP appears to represent a threshold required to induce higher deoxygenation and decreased tissue oxygenation levels within this form of resistance training. As importantly, we provide evidence that setting BFR to 80% of AOP exerts no further impact in altering microvascular deoxygenation compared to that seen using 60%. This is important because it corroborates past findings on BFR-induced muscle fatigue and activation (Fatela et al., 2016).

The use of different restrictive pressures interacts with muscle activation and neuromuscular fatigue (Loenneke et al., 2015; Fatela et al., 2016). According to our findings, this is also the case for microvascular oxygenation during acute low-intensity BFR exercise. BFR is thought to reduce venous return, while eliciting a turbulent flow in the arterial circulation. Ultimately, this decreases blood flow velocity within the tissues distal to the cuff (Manini and Clark, 2009). There is general agreement that the acute impact of BFR induces local hypoxia as well as an accumulation of metabolites (resulting from increased production and limited removal) (Scott et al., 2015). Our findings of increased deoxygenation and lower tissue O_2_ saturation, obtained during exercise with 60 and 80% AOP, further support this concept. This is the first study focusing on the interaction between BFR and tissue oxygenation, using a typical and well-validated exercise protocol (Loenneke et al., 2014a; Scott et al., 2015). Past reports have used NIRS measurements to examine muscle oxygenation during handgrip, eccentric, isometric or limited volume exercise as well as in response to muscle contractions performed to failure (Cayot et al., 2014; Ganesan et al., 2015; Lauver et al., 2017; Yanagisawa and Sanomura, 2017; Kilgas et al., 2018). Accordingly, comparisons between our results and those of the available research are challenging. Nevertheless, using an exercise protocol combining CON and ECC muscle contractions, Lauver et al. (2017), also showed heightened deoxygenation with BFR set at 130% of resting systolic blood pressure. Similar findings were also reported for low-volume isometric exercise, combined with BFR set at 60 and 80% of AOP (Cayot et al., 2014). Conversely, Ganesan et al. (2015) did not find a higher muscle oxygen extraction in BFRE knee extension exercise sets when compared with time matched unrestricted exercise. However, these authors prescribed an absolute and arbitrary BFR value of 100 mm Hg, which most likely affected their findings.

In our study, the differences observed in the Deoxy[Hb+Mb] response were noticeable before the beginning of exercise – a time point representing the effect of BFR on microvascular oxygenation *per se*. Using 60 and 80% AOP resulted in higher values of Deoxy[Hb+Mb] throughout the testing protocol. While Kilgas et al. (2018) showed a progressive increase in Deoxy[Hb+Mb] during exercise set at 60 and 80% AOP, we found that, under these specific circumstances, deoxy[Hb+Mb] remained unaltered from pre-exercise to the end of each set. Such discrepancies might be secondary to the differences between studies in the total length of time exposure to BFR (longer in our study). As importantly, Kilgas et al. (2018) analyzed only 1 set of isolated handgrip exercise for each restrictive pressure and all participants exercised using several levels of BFR within the same day. Thus, data from this study are most likely contaminated by the BFR-associated reactive hyperaemia (Mouser et al., 2017). However, we must acknowledge that we did not establish the level of venous return restriction induced by the different restrictive pressures. Therefore, we cannot establish on what extent the increased extraction in 60 and 80% AOP was due to the limited venous return.

We calculated the accumulated Deoxy[Hb+Mb] variation across the total exercise protocol. This represents the total oxygen extraction occurring during both exercise and rest periods (Soares et al., 2017). We found that the accumulated Deoxy[Hb+Mb] increased in parallel with BFR relative pressure. However, there were no differences between the impact of 60 and 80% AOP. Although we did not measure the pressure required to occlude venous return, we speculate that above 60% AOP there was no added effect; thus explaining the *plateau* on deoxy [HHb+Mb]. In fact, previous research shows that venous occlusion occurs at somewhat lower pressures. For example, it may be induced at an absolute pressure of 60 mmHg or even at 20 mmHg below diastolic blood pressure. Moreover, it has also been shown that BFR pressures between 45 and 200 mmHg produce a similar effect on reducing left ventricular end-diastolic volume – thus corroborating the concept that venous occlusion is attained at low levels of cuff pressure (Iida et al., 2005; Corrigan et al., 2008). In support of this, in most studies focusing on measuring blood flow to the lower limb using plethysmography, 50 mmHg is generally used as effective for inducing total venous occlusion (Patterson and Ferguson, 2010), and this is considerably lower than the pressure used at 60% AOP. In fact, the pressure that we used to induce 60% AOP was > than 50 mmHg and > than diastolic blood pressure. Conceptually, this confirms that there is no added restriction of venous return between 60 and 80% AOP. Additionally, we found that in the recovery periods between sets, the two higher pressures presented significantly higher values of T[HHb+Mb], which can be considered a proxy for blood flow. However, there were no differences between 60 and 80% AOP in this variable.

It has been hypothesized that the optimal BFR pressure may follow a hormetic-like relationship (Loenneke et al., 2014b; Scott et al., 2015; Kilgas et al., 2018), with some literature postulating that high pressures may not enhance muscular development more than moderate pressures. Even though recent data suggest that BFR exercise protocols may benefit from higher levels of restriction (80%) when exercising at very low intensities (Lixandrão et al., 2015), the authors only compared the effects of 12 weeks of training with either 40 or 80% AOP. Our data are in accordance with those of Kilgas et al. (2018) for arm exercise in that 60% AOP might represent a physiological threshold for tissue hypoxia and metabolite accumulation during low-intensity exercise.

[T(Hb+Mb)], which is considered as a surrogate index of changes in tissue blood volume (Grassi et al., 1999; Van Beekvelt et al., 2001; Perrey and Ferrari, 2018), was also influenced by different levels of restrictive pressures before exercise. Specifically, heightened restrictive pressures induced progressive increases in [T(Hb+Mb)], which can be attributed to the venous pooling distal to the cuff (Pope et al., 2013; Lauver et al., 2017). However, at a BFR level > than 60% of AOP we observed no further increase in [T(Hb+Mb)], which could result from similar degrees of venous return constrains in these two restrictive pressures. However, it is important to note that 80% AOP was the only condition compatible with a lower TOI and a small, but significant decrease in peak torque post-exercise. Since Deoxy[Hb+Mb] and [T(Hb+Mb)] were similar between 60 and 80% AOP, the greater magnitude of reduction in TOI values at higher levels of relative restriction was most likely caused by further reductions in arterial inflow.

In contrast to that seen during exercise, the inter-set recovery periods were characterized by considerable differences between conditions even at higher percentual of AOP. Globally, our data indicate that recovery of muscle deoxygenation resulting from each set of contractions varies depending on the magnitude of restrictive pressure. Specifically, while the relative recovery of Deoxy[Hb+Mb] was similar between NOBFR and 40% AOP, considerable differences were obtained when comparing 60 to 80% AOP. Specifically, we found that after low-intensity exercise performed at 60% AOP, Deoxy[Hb+Mb] does not recovery after 30 s of pause between sets. Conversely, when exercising at 80% AOP, muscle deoxygenation was actually potentiated during each inter-set rest interval. Although beyond the scope of this study, we speculate that this might be secondary to enhancements in the O_2_ deficit resulting from exercise performed at higher levels of BFR (Mendonca et al., 2015; Conceição et al., 2018).

According to the existent literature, the metabolic stress induced from multiple sets of BFR exercise is similar to that seen during high-intensity exercise, but only if the cuff inflation is maintained during recovery from each set (Suga et al., 2012). This approach ensures heightened muscle activation and maximizes training adaptations, independently of the external load (Loenneke et al., 2011). Although we did not compare this setting with a protocol where the cuff was release between sets, our results seems to be in line with the literature. However, we showed that all conditions were compatible with some recovery in tissue oxygenation during each inter-set period. Yet, there was a more pronounced recovery of TOI in NOBFR and 40% AOP compared to that seen during 60 and 80% AOP. Thus, we contend that, for ensuring metabolite buildup during exercise, the cuff should be kept inflated at a level ≥60% AOP.

### Limitations

There are several limitations in this study. For instance, the contribution of myoglobin desaturation is not possible to determine via NIRS. Thus, the differences between blood-muscle oxygen transport are not distinguishable in the current study (Ferrari et al., 2011). Additionally, NIRS signal is influenced by the thickness of adipose tissue and by the effect of changes in blood volume along within the tissue (Ferrari et al., 2004). Furthermore, even though we took precautionary measures to improve the reproducibility of probe placement (i.e., using indelible ink and taking photographic records), the day-to-day variation of these specific parameters corresponds to ∼ 8.0–9.4% (Willis et al., 2017). Nevertheless, we are confident that our data were not affected by this, because the use of relative changes in the NIRS variables, which is common in the research area, allows valid comparisons between settings (Perrey and Ferrari, 2018).

We did not establish the venous return occlusion pressure nor the deoxygenation response in full arterial occlusion, which could have provided a better understanding of the similarities between 60 and 80% AOP.

## Conclusion

Our findings indicate that a relative pressure above 40% of the AOP at rest seems to be required to potentiate the metabolic stress of low-intensity knee extension exercise. Second, they also provide evidence that with the cuff inflated between sets, there is a hampered recovery of tissue oxygenation and deoxygenation levels between higher and lower BFR pressures. Third, they also demonstrate that BFR ≥ 60% of AOP does not enhance oxygen extraction during low-intensity exercise. Fourth, we found an interaction between the magnitude of BFR (from 60 to 80%) and the recovery of muscle deoxygenation during the inter-set rest intervals. Thus, while deoxygenated [HHb+Mb] remains virtually unchanged during recovery from each set of exercise performed at 60% AOP, its values actually increase when using 80% AOP.

## Ethics Statement

This study was approved by the Faculty’s Ethics Committee (CEFMH 17/2014) and in accordance with the Declaration of Helsinki.

## Author Contributions

JR, PF, GM, MV, PM-H, and FA conceived and designed the experiments. JR, PF, MV, and JI performed the experiments. JR, PF, JV, JI, and FA analyzed the data. JR, PF, JV, PM-H, and FA interpreted results of research. JR, JV, MV, and PF drafted the manuscript and prepared the tables and figures. JR, PF, GM, JV, MV, JI, PM-H, and FA edited, critically revised the manuscript and approved the final version of manuscript.

## Conflict of Interest Statement

The authors declare that the research was conducted in the absence of any commercial or financial relationships that could be construed as a potential conflict of interest.
